# Mutations of the Mouse ELMO Domain Containing 1 Gene (*Elmod1*) Link Small GTPase Signaling to Actin Cytoskeleton Dynamics in Hair Cell Stereocilia

**DOI:** 10.1371/journal.pone.0036074

**Published:** 2012-04-27

**Authors:** Kenneth R. Johnson, Chantal M. Longo-Guess, Leona H. Gagnon

**Affiliations:** The Jackson Laboratory, Bar Harbor, Maine, United States of America; University of Tokyo, Japan

## Abstract

Stereocilia, the modified microvilli projecting from the apical surfaces of the sensory hair cells of the inner ear, are essential to the mechanoelectrical transduction process underlying hearing and balance. The actin-filled stereocilia on each hair cell are tethered together by fibrous links to form a highly patterned hair bundle. Although many structural components of hair bundles have been identified, little is known about the signaling mechanisms that regulate their development, morphology, and maintenance. Here, we describe two naturally occurring, allelic mutations that result in hearing and balance deficits in mice, named roundabout (*rda*) and roundabout-2J (*rda^2J^*). Positional cloning identified both as mutations of the mouse ELMO domain containing 1 gene (*Elmod1*), a poorly characterized gene with no previously reported mutant phenotypes. The *rda* mutation is a 138 kb deletion that includes exons 1–5 of *Elmod1*, and *rda^2J^* is an intragenic duplication of exons 3–8 of *Elmod1*. The deafness associated with these mutations is caused by cochlear hair cell dysfunction, as indicated by conspicuous elongations and fusions of inner hair cell stereocilia and progressive degeneration of outer hair cell stereocilia. Mammalian ELMO-family proteins are known to be involved in complexes that activate small GTPases to regulate the actin cytoskeleton during phagocytosis and cell migration. ELMOD1 and ELMOD2 recently were shown to function as GTPase-activating proteins (GAPs) for the Arf family of small G proteins. Our finding connecting ELMOD1 deficiencies with stereocilia dysmorphologies thus establishes a link between the Ras superfamily of small regulatory GTPases and the actin cytoskeleton dynamics of hair cell stereocilia.

## Introduction

Many medically important hearing and balance disorders are caused by defects in the sensory hair cells of the inner ear. Modified microvilli called stereocilia project from the apical surfaces of hair cells and are tethered together by fibrous links to form hair bundles. Hair bundle deflections, caused by fluid movements induced by sound pressure or head movements, open mechanically gated transduction channels located at the stereocilia tips [1]. The resulting influx of cations depolarizes the hair cell and triggers neurotransmitter release, which initiates the transfer of auditory or vestibular information to the brain. Hair bundles thus are essential to the mechanoelectrical transduction process, and proper development and maintenance of these key structures is critical for normal hearing and balance.

Each hair cell has a single hair bundle composed of tens to hundreds of stereocilia that are arranged in precise rows of increasing height, forming a uniform staircase pattern. Each stereocilium consists of a core of actin filaments surrounded by the plasma membrane. Molecular genetic analyses of human and mouse deafness mutations and the analysis of hair-bundle proteins have identified several constituents of the hair bundle and have provided insight into its development and function [2], [3], [4], [5]. In addition to the cytoskeletal proteins associated with its actin core, stereocilia contain motor proteins, scaffolding proteins, adhesion proteins, integral membrane proteins, calcium regulatory proteins, and proteins involved in ATP synthesis. Protein complexes forming stereociliary links are particularly important in the development and cohesion of the hair bundle [6], [7].

Although much progress has been made in identifying the structural components of the hair bundle [8], little is known about the signaling pathways that regulate hair bundle development. Small guanosine triphosphatases (GTPases) of the Rho and Arf families are key regulators of actin cytoskeleton dynamics in many cell types and species [9], [10]. Several lines of evidence suggest that small GTPases also may be involved in regulating the actin dynamics of hair cells [11], [12], [13]. For example, diaphanous proteins can act as effectors for Rho GTPases to regulate actin polymerization [14], [15], and a mutation of the human *DIAPH1* gene underlies the nonsyndromic deafness disorder DFNA1 [16]. Recently, an analysis of late stage mouse embryos with an inner ear-specific knockout of the *Rac1* gene demonstrated an essential role for this Rho-family GTPase in cochlear development and hair cell morphogenesis [13].

The mammalian engulfment and cell motility protein ELMO1 can form a complex with Dock proteins to activate Rac1 and promote cytoskeletal reorganization [17], [18]. In *C. elegans*, CED-12 (an ortholog of ELMO1) interacts with CED-5 (an ortholog of Dock180) to activate CED-10 (an ortholog of Rac1) and promote cytoskeletal rearrangements [19], [20], indicating that this signaling pathway is evolutionarily conserved. In mammals, six proteins have been identified that contain the conserved ELMO domain (Prosite PDOC51335): ELMO1, ELMO2, and ELMO3 (orthologs of *C. elegans* CED-12) and three more distantly related proteins – ELMODl, ELMOD2, and ELMOD3 [21]. While ELMO1, ELMO2, and ELMO3 are known to act as guanine nucleotide exchange factors (GEFs) to activate Rac1 [19], ELMOD1 and ELMOD2 were shown to function as GTPase-activating proteins (GAPs) for the Arf family of small G proteins [22]. GEFs and GAPs serve as molecular switches for small G proteins. When in their inactive GDP-bound form, small regulatory GTPases can be activated by GEFs, which replace the bound GDP with free GTP, and when in their active GTP-bound form, they can be inactivated by GAPs, which increase the rate of hydrolysis of GTP to GDP.

Here, we describe two independent spontaneous inactivating mutations of the mouse *Elmod1* gene that cause deafness and balance defects. The inner ear dysfunction caused by these mutations is associated with dysmorphic hair cell stereocilia and implicates ELMOD1 in a signaling pathway that regulates the maturation and stability of these actin-based structures. In particular, the stereocilia of inner hair cells become greatly elongated and fused in ELMOD1 deficient mice. The *Elmod1* mutations described here thus provide the first evidence for the involvement of small GTPases in regulation of hair bundle maturation.

## Results

### Phenotypes of the allelic *rda* and *rda^2J^* mutations

Both roundabout (*rda*) and roundabout-2 Jackson (*rda^2J^*) mutations, when homozygous, cause circling behavior and loss of acoustic startle response in mice, phenotypes consistent with auditory and vestibular hair cell degeneration. Both mutations mapped to the same region of Chromosome (Chr) 9. We analyzed 123 mutant (*rda/rda*) F2 progeny from an intercross of (C57BL/6J-*rda* X CAST/EiJ) F1 hybrids and mapped the *rda* mutation between markers *D9Mit231* and *D9Mit248*, an interval of 6.12 mega bases (Mb), corresponding to the 52.09–58.21 Mb region of Chr 9 (NCBI Build 37). We analyzed 79 mutant (*rda^2J^/rda^2J^*) mice from an intercross of (B6.Cg-*rda^2J^* X CAST/EiJ) F1 hybrids and mapped the *rda^2J^* mutation between markers *D9Mit231* and *D9Mit141*, a 5.82 Mb interval (52.09–57.91 Mb region of Chr 9). Because of their nearly identical phenotypes and genetic map positions, the *rda* and *rda^2J^* mutations were tested for allelism. Separate matings of two +/*rda* females with two *rda^2J^/rda^2J^* males produced four litters consisting of 12 mutant and 11 non-mutant progeny. The presence of mutant progeny in the expected 1∶1 Mendelian ratio indicated non-complementation and confirmed that the two mutations are allelic.

**Figure 1:**
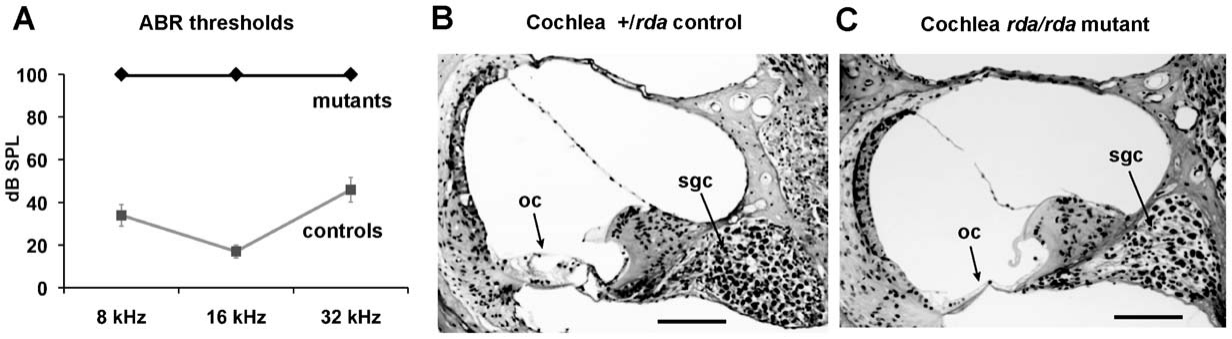
Deafness and cochlear pathology of *rda* and *rda^2J^* mutant mice. **A.** ABR thresholds (dB SPL) of *rda/rda* and *rda ^2J^/rda^2J^* mutant mice and non-mutant controls tested at 33–48 days of age. ABRs for 8, 16, and 32 kHz stimulus frequencies were not detected in any of the *rda/rda* (N = 9) and *rda^2J^/rda^2J^* (N = 8) mutant mice tested, even with the maximum stimulus presentation of 100 dB SPL. Heterozygous +/*rda* (N = 8) and +/*rda^2J^* (N = 4) mice and homozygous +/+ B6 mice (N = 17) were combined as controls because their ABR thresholds did not significantly differ from one another. Error bars represent standard deviations of the threshold means. **B, C.** Cross sections through the basal turn of the cochlea from a +/*rda* heterozygous control mouse (B) and a littermate *rda/rda* mutant mouse (C) examined at 4 months of age. Note the complete degeneration of the organ of Corti (oc) and decreased density of spiral ganglion cells (sgc) in the *rda/rda* cochlea. Scale bars represent 100 microns. Cochlear cross sections of *rda^2J^* mutant mice (not shown) exhibited this same pathology.

Hearing in *rda* and *rda^2J^* mutant mice and controls was further assessed by auditory brainstem response (ABR) measurements (Figure 1A). The lack of detectable ABRs for pure tone 8 kHz, 16 kHz, and 32 kHz stimuli, even at the maximum sound pressure level presented (100 dB), demonstrated that *rda/rda* and *rda^2J^/rda^2J^* mutant mice on the C57BL/6J (B6) strain background are profoundly deaf by 5 weeks of age - the youngest age tested. ABR thresholds of +/*rda* and +/*rda^2J^* heterozygotes were the same as +/+ controls of the B6 background strain. Cross sections through the basal turns of cochleae from mutant mice at 4 months of age showed a complete degeneration of the organ of Corti and partial loss of spiral ganglion cells (Figure 1B).

**Figure 2:**
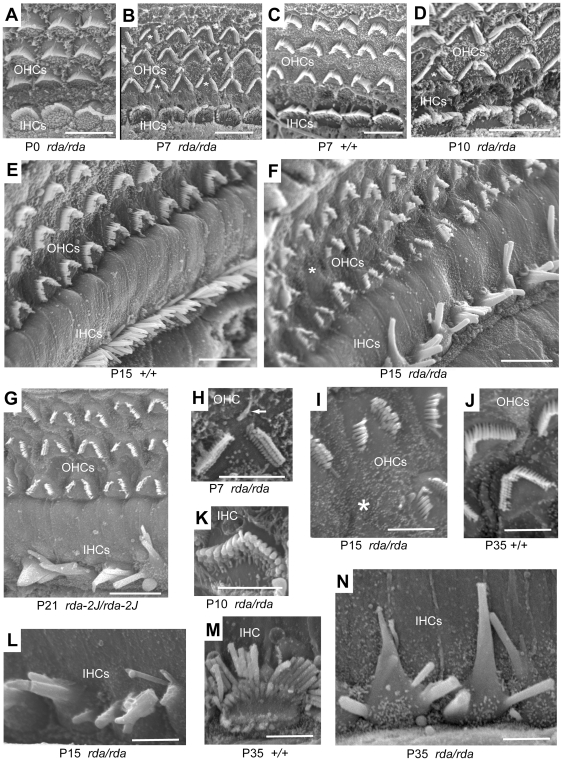
Cochlear hair cell abnormalities in *rda* and r*da^2J^* mutant mice. Examination of the apical surfaces of cochlear hair cells by scanning electron microscopy showed normal bundle morphology in both inner hair cells (IHC) and outer hair cells (OHC) of *rda/rda* mutant mice at P0 (**A**). At P7 the stereocilia adjacent to the kinocilium (marked by arrow in **H**) had degenerated in some of the OHC bundles (marked by asterisks in **B, D**) of *rda/rda* mice, compared with the fully intact bundles of wildtype (+/+) controls (**C**). IHC bundles of *rda/rda* mice retained a normal appearance at P10 (**D, K**), but by P15 (**F, L**) all IHCs of mutant mice exhibited stereocilia elongations and fusions as compared with the normal IHC bundle morphology of age-matched controls (**E**). IHCs at P35 illustrate the striking degree of stereocilia elongations and fusions seen in *rda/rda* mutants (**N**) compared with age-matched controls (**M**). In contrast to IHCs, the OHC stereocilia of *rda/rda* mice did not elongate or fuse, but they did degenerate over time. By P15, nearly all OHCs had lost stereocilia at the bundle peak and a few (marked by asterisks) had lost the entire bundle (**F, I**), as compared with the intact OHC bundles of control mice (**J**). The IHC and OHC bundle abnormalities of *rda^2J^/rda^2J^* mutant mice (**G**) were very similar to those of *rda/rda* mice (**F**). Scale bars: A–G, 10 microns; H–N, 5 microns. All hair cells shown in the figure are from the middle region of the cochlea, but similar bundle abnormalities were seen in other regions (not shown), consistent with the absence of an ABR at all test frequencies (Fig. 1A).

Because hair bundle dysmorphology often precedes hair cell death, we examined stereocilia of young mutant and control mice by scanning electron microscopy (SEM). Stereocilia bundle morphology of inner hair cells (IHC) and outer hair cells (OHC) appeared normal in newborn (P0) *rda/rda* mutant mice (Figure 2A), but abnormalities became apparent at older ages. At P7 and P10, a few OHCs of *rda/rda* mice exhibited abnormal hair bundles, wherein the stereocilia directly adjacent to the kinocilium had degenerated (Figure 2B, D, H), and by P15 all OHCs exhibited noticeable degrees of stereocilia degeneration with a few completely lacking bundles (Figure 2F, I). IHC bundle morphology appeared normal at P7 and P10 (Figure 2B, D, K), but by P15 a striking elongation and fusion of stereocilia was apparent in all IHCs (Figure 2F, L, N). SEM length estimates of the tallest stereocilia from medial turn inner hair cells of P15 and P35 mutants were 6–10 microns compared with 3–4 microns for non-mutant controls. Hair bundle abnormalities were similar in *rda^2J^* mutants (Figure 2G).

### High-resolution genetic mapping

To refine the map position of *rda* for positional cloning of the responsible gene, we produced a total of 1357 F2 mice from the intercross of (C57BL/6J-*rda* X CAST/EiJ) F1 hybrids. All F2 progeny produced from the linkage cross were genotyped at weaning for the *D9Mit231* and *D9Mit248* flanking markers, and 48 mice with informative recombinant chromosomes were further analyzed (Table S1). Of these recombinant mice, 30 had flanking markers with B6/CAST and B6/B6 genotypes and were evaluated for hearing impairment by ABR; 18 of these had significantly elevated ABR thresholds (>20 dB above normal) when tested at 4–6 weeks of age and were classified as *rda/rda* mutants; the other 12 mice had normal thresholds and were classified as +/*rda*. The 18 recombinant mice that had flanking markers with B6/CAST and CAST/CAST genotypes were progeny tested to distinguish between +/*rda* and +/+ genotypes.

ABR testing was used to unambiguously classify homozygous mutant mice because circling behavior of *rda/rda* mice in the F2 linkage cross was not fully penetrant. An analysis of non-recombinant, obligate *rda/rda* mice from the intercross showed that all exhibited elevated ABR thresholds, but only about 70% showed circling behavior. Although all *rda/rda* F2 mice exhibited significant hearing impairment, the degree of impairment was variable, indicating the influence of strain-specific modifier gene or genes.

DNA samples from recombinant mice were typed for additional markers within the original 6.12 Mb candidate interval, including microsatellite markers *D9Mit334* and *D9Mit100* and polymorphisms within the *Exph5*, *Atm*, and *Chrna5* genes. We developed four additional microsatellite markers by designing PCR primers that flank variable CA-repeats, which we identified from the reference genome sequence of C57BL/6J mice. Refined analysis of chromosome crossover positions in the recombinant mice, coupled with the deduced *rda* genotypes of these mice, enabled us to reduce the candidate gene interval to 1.14 Mb, between *Exph5* at the 53.186 Mb position and a CA repeat marker at 54.326 Mb. Details of recombinant genotypes used to determine the 1.14 Mb candidate gene interval for the *rda* mutation are shown in Table S1. The *rda* candidate region contained 16 annotated genes: *Kdelc2*, *4930550C14Rik*, *Atm*, *Npat*, *Acat1*, *Cul5*, *Rab39*, *n-R5s83*, *Slc35f2*, *Sln*, *Elmod1*, *Tnfaip8l3*, *Cyp19a1*, *1700104A03Rik*, *Gldn*, and *Dmxl2*. The ELMO domain containing 1 gene (*Elmod1*) at the 53.8 Mb position of Chr 9 was considered the most plausible candidate gene for *rda* because it is primarily expressed in neural tissues (UniGene Mm.259791), and an *ELMOD1* EST (AW022370.1) was identified in a human fetal cochlea library.

### *Elmod1* gene structure and expression

**Figure 3:**
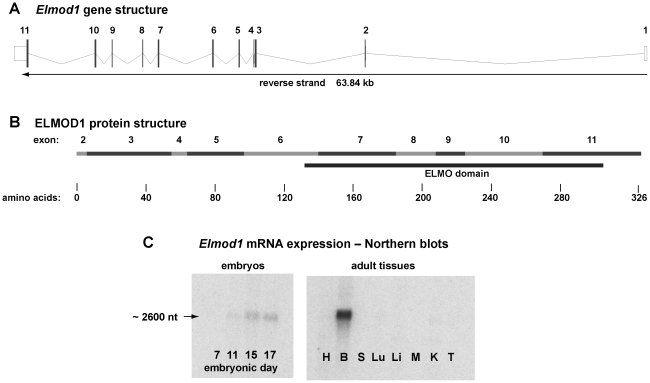
*Elmod1* gene structure and expression. **A.** The mouse *Elmod1* gene spans 63.84 kb and is comprised of 11 exons transcribed on the reverse strand of the NCBI genomic DNA reference sequence (Chr 9: 53,823,108 to 53,759,267 bp, Build 37). **B.** The 2605 nt reference mRNA sequence (NM_177769) encodes a 326 amino acid protein (NP_808437). Amino acids 132–305 (encoded by exons 6–11) comprise a conserved ELMO/CED-12 domain (pfam04727, IPR006816) characteristic of the ELMO protein family. Diagrams A and B were downloaded from the Ensembl website (http://www.ensembl.org/). **C.** *Elmod1* gene expression was examined by northern blot analysis. Commercially prepared blots from mouse embryos and adult tissues (MTN blots, Clontech, Palo Alto, CA) were hybridized with a mouse *Elmod1* cDNA probe corresponding to nucleotides 426–904 of the NM_177769 reference cDNA sequence. Blots contained purified Poly A+ RNA from 7 day (lane 1), 11 day (lane 2), 15 day (lane 3) and 17 day (lane 4) embryos and from heart (H), brain (B), spleen (S), lung (Lu), liver (Li), skeletal muscle (M), kidney (K), and testis (T) of adult mice. A single transcript of about 2,600 nucleotides was detected, most highly expressed in adult brain.

The 2605-nucleotide reference sequence for mouse *Elmod1* mRNA (NM_177769) is transcribed from 11 exons and encodes a 326 amino acid protein (reference sequence NP_808437). We found that the predominant *Elmod1* transcript in adult mouse brain corresponds with this reference sequence and its derivative exon structure (Figure 3A). Detailed results from our analyses of alternative transcripts and determination of the transcription start site of *Elmod1* are presented in Figure S1. The protein encoded by *Elmod1* (Fig. 3B) contains a conserved domain (ELMO/CED-12) that is characteristic of the ELMO family of proteins, whose members mediate the activation or inactivation of small G proteins that regulate actin cytoskeleton dynamics [19], [21], [23]. Dysregulation of actin-based hair cell stereocilia formation or maintenance caused by ELMOD1 deficiency might, therefore, account for the hair bundle abnormalities observed in *rda* and *rda^2J^* mutant mice (Figure 2).

Northern blot analysis of wildtype mice (Figure 3C) showed that *Elmod1* expression is barely detectable in 11-day embryos but progressively increases in 15 and 17 day-old embryos. *Elmod1* is highly expressed in wildtype adult brain with low or undetectable expression in heart, spleen, lungs, liver, skeletal muscle, kidney, and testis. A single ∼2600 nucleotide transcript was detected in RNA from embryos and adult brain. Though not quantitative, RT-PCR showed that *Elmod1* is also expressed in tissues of the adult inner ear.

### Molecular analysis of the *rda* mutation of *Elmod1*

**Figure 4:**
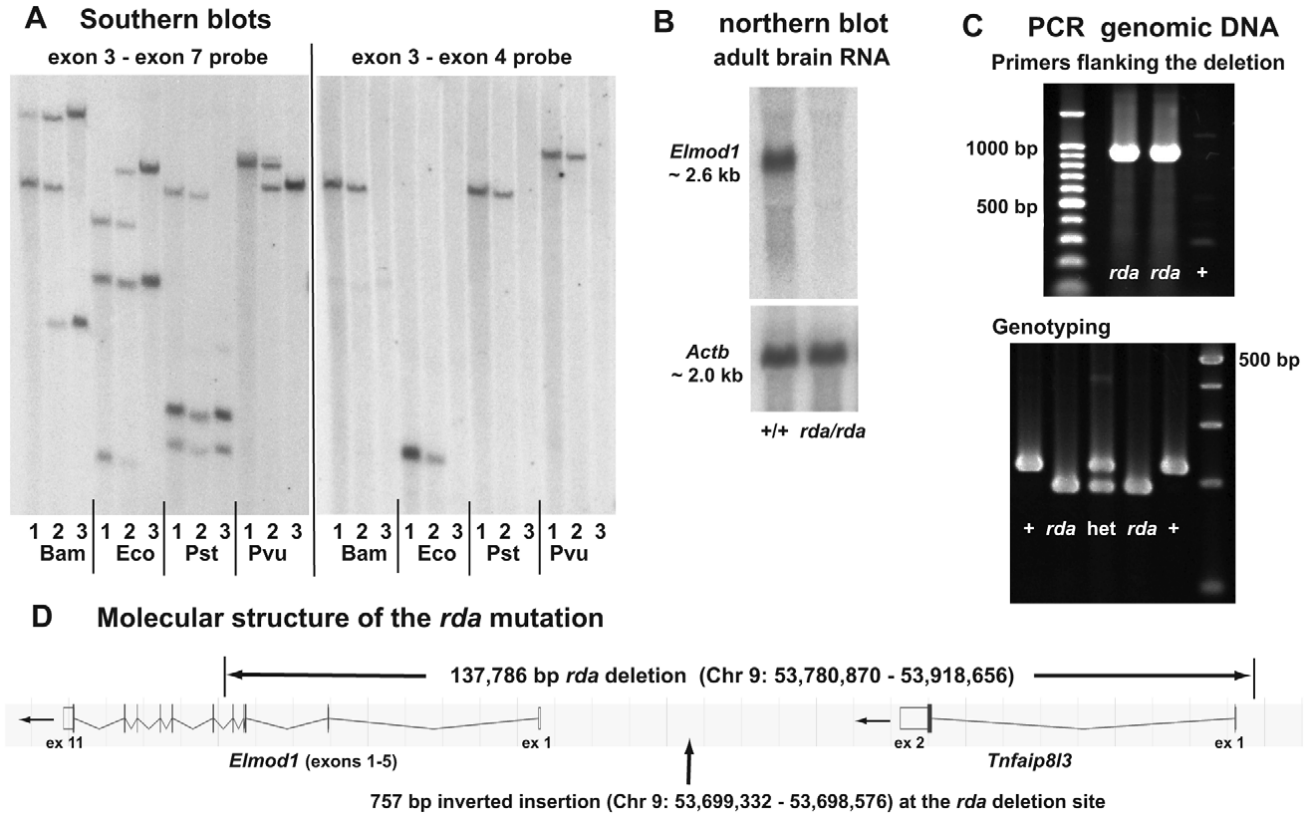
Molecular analysis of the E*lmod1^rda^* mutation. Exons 1–5 of *Elmod1* are deleted in *rda* mutant chromosomes. **A**. Southern blots of genomic DNA from mice with +/+ (lanes marked 1), +/*rda* (lanes marked 2) and *rda/rda* (lanes marked 3) genotypes, digested with *BamH*I (Bam), *EcoR*I (Eco), *Pst*I (Pst), and *Pvu*II (Pvu) restriction enzymes. The blots shown were hybridized with *Elmod1* cDNA probes corresponding to exons 3–7 (nucleotides 426–904 of NM_177769, blot on left) and exons 3–4 (nucleotides of NM_177769,blot on right). *Elmod1* mRNA is not expressed in *rda/rda* mutant mice. **B**. Northern blots of total RNA extracted from brains of adult C57BL/6J controls (+/+) and *rda/rda* mutants was hybridized first with an *Elmod1* cDNA probe (nucleotides 426–904 of NM_177769, top figure) and subsequently with a beta actin control probe (*Actb*, bottom) to evaluate RNA loading concentrations. **C**. PCR with primer pairs spanning the genomic region around *Elmod1* was used to define the extent of the *rda* deletion. Primers immediately flanking the mutation amplified a 963 bp product in mutant (*rda*) but not wildtype (+) DNA, and sequencing of this product identified a 757 bp insertion. A three-primer assay was developed to distinguish homozygous wildtype mice (+), *rda*/+ heterozygotes (het), and *rda/rda* homozygous mutants (*rda*). **D**. The molecular structure of the *rda* mutation was precisely defined as a 137,786 bp deletion with a 757 bp inverted insertion at the site of the deletion. Exons 1–5 of *Elmod1* and the entire *Tnfaip8l3* gene are included in the deletion.

PCR amplification of genomic DNA from *rda/rda* mutant mice with primers flanking individual *Elmod1* exons (Table S3A) revealed a deletion of exons 1–5. Southern blot analysis with different *Elmod1* cDNA probes confirmed this partial deletion, showing missing restriction fragments in genomic DNA from *rda/rda* mice that corresponded with the exon 1–5 region of the gene (Figure 4A). Northern blot analysis showed a complete loss of *Elmod1* expression in *rda/rda* mutant mice (Figure 4B).

The extent of the *rda* deletion was refined by PCR analysis with 27 additional primer pairs distributed throughout the genomic region surrounding the *Elmod1*gene. The presence or absence of PCR products delimited the extent of the deleted region. A 963 bp PCR product was amplified in *rda/rda* DNA using primers closely flanking the deleted region (Figure 4C, Table S3B), and sequence analysis of this PCR product revealed a deletion of 137,786 bp, corresponding to the 53,780,870 to 53,918,656 bp region of Chr 9. DNA sequence analysis also revealed a 757 bp insertion at the site of the deletion that corresponds to the inverted 53,699,332–53,698,576 bp region of Chr 9, originating from within the single intron of the sarcolipin gene (*Sln*).

The *rda* deletion includes exons 1–5 of the *Elmod1* gene, which is transcribed on the reverse strand from 53,823,108 to 53,759,267 bp. The *rda* deletion also includes the tumor necrosis factor, alpha-induced protein 8-like 3 gene (*Tnfaip8l3*), which also is transcribed from the reverse strand (53,916,214 to 53,873,415 bp). *Tnfaip8l3* is an uncharacterized gene with unknown function. A three-primer assay (Figure 4C, Table S3B) was developed to distinguish +/+, +/*rda*, and *rda/rda* genotypes. The molecular structure of the *rda* mutation is illustrated in Figure 4D.

### Molecular analysis of the *rda^2J^* mutation of *Elmod1*

**Figure 5:**
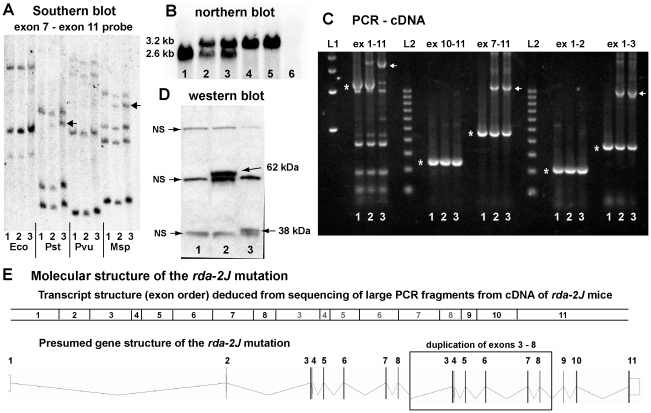
Molecular analysis of the E*lmod1^rda-2J^* mutation. **A.** Southern blots of genomic DNA from mice with +/+ (lanes marked 1), +/*rda^2J^* (lanes marked 2) and *rda^2J^/rda^2J^* (lanes marked 3) genotypes, digested with *Eco*RI (Eco), *Pst*I (Pst), *Pvu*II (Pvu), and *Msp*I (Msp) restriction enzymes. The blot was hybridized with an *Elmod1* cDNA probe corresponding to exons 7–11. Although the same quantity of DNA was loaded in each lane, the intensity of *Elmod1*-hybridizing bands is greater in in *Eco*RI- and *Pvu*II-digested DNA samples from *rda^2J^/rda^2J^* mice than samples from +/+ mice and exhibit additional bands (indicated by arrows) in *Pst*I and *Msp*I digested DNA. **B.** A northern blot of total RNA extracted from brains of adult C57BL/6J control (lane 1), *rda^2J^*/+ heterozygotes (lanes 2 and 3), *rda^2J^/rda^2J^* homozygotes (lanes 4 and 5), and *rda/rda* homozygote (lane 6; negative control) was hybridized with an *Elmod1* cDNA probe. Wildtype *Elmod1* transcript (∼2600 nt) was not detected in RNA from *rda^2J^/rda^2J^* mice; however, a 3200 nt mutant transcript (about 600 nucleotides larger than wildtype) was abundantly expressed. **C.** PCR products from *rda^2J^*–derived cDNAs indicate an intragenic duplication. cDNAs from mice with +/+ (lanes marked 1), +/*rda^2J^* (lanes marked 2) and *rda^2J^/rda^2J^* (lanes marked 3) genotypes were used as PCR templates in combination with primers specific to *Elmod1* exons. Expected wildtype PCR products are marked by asterisks, and unexpected large PCR products unique to *rda^2J^* samples are indicated by arrows. 500 bp (lane marked L1) and 100 bp (lane marked L2) ladders were used to estimate PCR product sizes, and predicted sizes are given in Table S2B. **D.** A western blot of protein extracts from adult brains of *rda/rda* (lane 1, negative control), *rda^2J^/rda^2J^* (lane 2), and +/+ B6 mice (lane 3), shows that the predicted 38 kDa wildtype ELMOD1 protein is absent from both *rda* and *rda^2J^* mutant mice, but a larger 62 kDa mutant protein can be seen in *rda^2J^* mutants (indicated by arrow in lane 2), corresponding to the predicted duplication of 202 amino acids. The polyclonal ELMOD1 antibody cross-reacted with other unknown proteins (non-specific bands, NS). **E.** The large PCR products obtained from *rda^2J^*–derived cDNA (indicated by arrows in **C**) and multiple combinations of exon-specific primers were used to determine the DNA sequence and structure of the mutant *Elmod1* transcript, and this analysis indicated a duplication of exons 3–8, as shown in the diagram of the presumed mutant gene structure.

Because *rda^2J^* was shown to be allelic with *rda*, we examined the *Elmod1* gene in *rda^2J^/rda^2J^* mice. The exons and adjacent splice sites of *Elmod1* were amplified from genomic DNA with PCR primers flanking individual exons (Table S3A), but no DNA sequence differences were found between the products of *rda^2J^* mutants and wildtype mice. Southern blot analysis with *Elmod1* cDNA probes, however, indicated some type of genomic rearrangement in *rda^2J^* DNA compared with wildtype DNA (Figure 5A). Northern blot analysis of brain RNA with an *Elmod1* cDNA probe showed that *rda^2J^* mutant transcripts (∼3.2 kb) were abundantly expressed but were much larger than wildtype transcripts (∼2.6 kb), and that no wildtype transcripts were produced in *rda^2J^/rda^2J^* mutant mice (Figure 5B).

PCR analysis of brain-derived cDNAs using primers corresponding to different exons of the *Elmod1* gene (Table S2) revealed the presence of extra, large amplification products in cDNAs of +/*rda^2J^* and *rda^2J^*/*rda^2J^* but not +/+ mice (Figure 5C). Not all of the combinations of exon-specific primers produced additional PCR products in mutant cDNAs, indicating that *Elmod1* is only partially duplicated. Sequence analysis of the large PCR products obtained from *rda^2J^*/*rda^2J^* cDNA with multiple combinations of *Elmod1* exon-specific primers indicated that the *rda^2J^* mutant transcript contains a 606 nt intragenic duplication of exons 3–8, which corresponds to a predicted 11.5 to 25.5 kb duplication in genomic DNA. The altered transcript and intragenic duplication of the *Elmod1^rda-2J^* mutation is illustrated in Figure 5E.

The 606 nt duplication in the *rda^2J^* transcript is in-frame and predicted to encode a 202 amino acid duplication, which is supported by western blot results (Figure 5D). The estimated mass of the wildtype protein is about 36 kDa, consistent with its predicted size of 326 amino acids, and the estimated mass of the *rda^2J^* mutant protein is about 62 kDa, consistent with its predicted size of 528 amino acids. Western blot results also demonstrate the absence of wildtype ELMOD1 protein in both *rda/rda* and *rda^2J^*/*rda^2J^*mutant mice (Figure 5D).

Because the *Tnfaip813* gene, as well as *Elmod1*, was deleted by the *rda* mutation, we examined DNA from *rda^2J^* mutant mice for possible alterations of *Tnfaip813*, although the spontaneous occurrence and coisogenic nature of the *rda^2J^* mutation would argue against any additional gene mutations. As expected, we found no evidence for a *Tnfaip813* mutation in *rda^2J^*/*rda^2J^* mutant mice; there were no exon, splice site, or cDNA sequence differences between wildtype controls and mutant mice. We conclude, therefore, that the common phenotype of *rda* and *rda^2J^* mutant mice is solely the result of *Elmod1* inactivating mutations.

### Cellular localization of *Elmod1* expression

**Figure 6:**
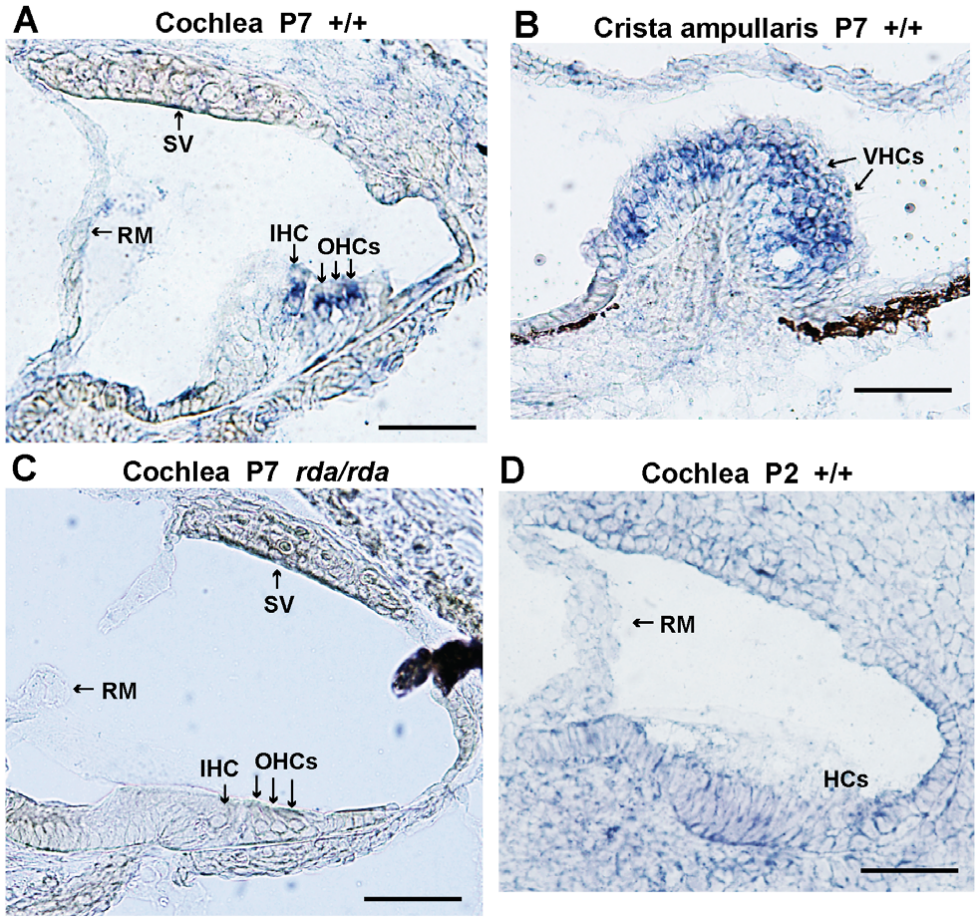
*In situ* expression of *Elmod1* mRNA in the inner ear. Expression was detected in inner hair cells (IHC) and outer hair cells (OHCs) of the cochlea (**A**) and vestibular hair cells (VHCs) of the crista ampullaris (**B**) in inner ears from wildtype (+/+) mice at postnatal day 7 (P7). Mutant *rda/rda* mice served as negative controls for probe specificity, as seen by the lack of detectable *Elmod1* expression in cochlear hair cells of P7 *rda/rda* mice (**C**). *Elmod1* expression was not detected above background staining in inner ears of P2 wildtype mice (**D**) or in P15 mice (not shown). The general location of hair cells (HCs) is indicated in the P2 cochlea (D), because inner and outer hair cells are not clearly distinguishable in cryosections at this age. For reference, the Reissner's membrane (RM) and stria vascularis (SV) of the cochleae are indicated with arrows. All panels are at the same magnification; the scale bar represents 50 micrometers.

We localized *Elmod1* mRNA expression by *in situ* hybridization with an antisense RNA probe. Using tissues of *rda/rda* mutant mice as negative controls, we confirmed *Elmod1*-specific expression in cells of the inner ear, brain, and eyes of wildtype mice at various time points. In the inner ears of postnatal day 7 (P7) mice, *Elmod1-*specific expression was restricted to cochlear and vestibular hair cells (Figure 6A, B). Inner ear expression was not detected above background staining levels in younger mice examined at E14.5 (not shown) and P2 (Figure 6D) nor in older mice examined at P15 (not shown). In the brain, *Elmod1*-specific expression was detected in Purkinje cells of the cerebellum and in granule cell and pyramidal neurons of the hippocampus examined at P7 and P15 (not shown) and at P45 (Figure S2A, B). Expression also was detected in retinal ganglion cells of the eye examined at P2 (Figure S2C) and at P15 (not shown). Although *Elmod1* appears to be highly expressed in these non-otic neural tissues, we detected no overt cellular abnormalities or degeneration in *rda/rda* mutant brains examined at 10 months of age. Electroretinography (ERG) tests of both *rda/rda* (tested at 3 months of age) and *rda^2J^/rda^2J^* (tested at 4 months of age) mutant mice showed normal retinal function.

Polyclonal antibodies to a recombinant ELMOD1 protein detected an ELMOD1-specific band in western blots; however, it also detected additional non-specific bands (Figure 5D). Because of this cross-reactivity, the antibodies could not be used to localize sub-cellular ELMOD1-specific protein expression in tissue sections.

## Discussion

### *Elmod1* mutations and hair bundle abnormalities

We used positional cloning to identify two independent, naturally occurring mutations of *Elmod1*, designated *rda* and *rda^2J^*. Both are complex DNA alterations; *rda* is a large 138 kb deletion with a small 756 bp inverted insertion at the point of the deletion, and *rda^2J^* is an in-frame intragenic duplication of exons 3–8. The *rda* deletion includes exons 1–5 of *Elmod1*, and as expected no *Elmod1* gene products (either mRNA or protein) were detected in *rda/rda* mutant mice. Although wildtype ELMOD1 protein is absent in *rda^2J^/rda^2J^* mice, a larger mutant form of the ELMOD1 protein is produced; however, the extensive duplication of 202 amino acids in the mutant protein likely makes it nonfunctional. The similarity of the *rda^2J^/rda^2J^* mutant phenotype to that of *rda/rda* mice provides additional evidence that *rda^2J^* is a null allele. That two independent, complex DNA alterations like *rda* and *rda^2J^* occur at the same gene locus seems unusual, but there is some evidence that structural variants have a nonrandom genomic distribution, and that multiple independent mutations may arise in close proximity, perhaps at unstable genomic regions [24].

We found that *Elmod1* expression localized to both cochlear and vestibular hair cells of the inner ear (Fig. 6), consistent with hair cell dysfunction being the underlying cause for the deafness and balance dysfunction of *Elmod1* mutant mice. In support of our *Elmod1* localization results, a recent study of cell type-specific transcriptomes from inner ear tissues of newborn mice identified *Elmod1* as one of a large cluster of genes that is differentially expressed in auditory and vestibular sensory epithelia [25]. *Elmod1* is expressed in vestibular hair cells of the crista ampullaris (Fig. 6B), which sense rotational movements of the head, thus their dysfunction could explain the hyperactive circling behavior of the *Elmod1* mutant mice. *Elmod1* is also expressed in Purkinje cells of the cerebellum (Fig. S2A). Cerebellar dysfunction in mice impairs balance and coordination and can result in ataxic behaviors that are similar to those caused by vestibular defects of the inner ear. It is possible that cerebellar function is compromised in *Elmod1* mutant mice but that the behavioral evidence for it is masked by the overlapping phenotype of inner ear dysfunction; however, we found no overt cellular abnormalities or degeneration of Purkinje cells in mutant mice. Likewise, we found no evidence of retinal ganglion cell degeneration or eye dysfunction in *Elmod1* mutant mice, although *Elmod1* is expressed in retinal ganglion cells (Fig. S2C). More refined structural and functional analyses of these non-otic *Elmod1*-expressing cells might reveal subtle abnormalities in mutant mice.

*Elmod1* encodes a member of the ELMO family of effector molecules for small GTPases, many of which are known to regulate actin cytoskeleton organization [21]. The hair bundle phenotype of *Elmod1* mutant mice suggests a similar role for ELMOD1 in regulating the actin dynamics that determine stereocilia length in mammalian inner hair cells. The concept that hair cell stereocilia exhibit a rapid treadmilling of their actin filament cores [26] and that the balance between actin polymerization and disassembly maintains the steady-state length of stereocilia [27], [28] has recently been put in doubt. New evidence indicates that stereocilia are remarkably stable with rapid turnover occurring only at the tips and not by a treadmilling process [29]. Thus, stereocilia lengths are determined during hair bundle maturation and once formed do not require a continuous steady-state maintenance. The analyses of mouse deafness mutations have identified several proteins involved in the regulation of hair cell stereocilia length, including espin [30], [31], myosin XVa [32], whirlin [33], [34], [35], and EPS8 [36], [37], whose absence results in shortened stereocilia, and myosin VIIa [38], myosin VI [39], [40] and PTPRQ [41], [42] whose absence results in elongated stereocilia. Mice lacking gelsolin also show elongated stereocilia, but only in outer hair cells at the cochlear apex [43]. The striking stereocilia elongations and fusions we observed in *Elmod1* mutant mice (Fig. 2) are more extensive than those reported in other mutant mice [36]–[41], and in contrast to these other mutants, are limited to cochlear inner hair cells.

Measurements of stereocilia length during development in mice reveal that the tallest stereocilia row of cochlear inner hair cells elongates by 60% from P7 to P13 [44]. This period of rapid stereocilia elongation, which occurs during normal hair bundle maturation, coincides with the onset time of the abnormal over-elongations and fusions of inner hair cell stereocilia observed in *Elmod1* mutant mice between P10 and P15. It also coincides with our *in situ* hybridization results, where *Elmod1* expression in hair cells could be detected at P7 but not at P2 or P15. ELMOD1 thus may be involved in a regulatory process that establishes the mature staircase architecture of the inner hair cell bundle. The actin-capping protein twinfilin 2 is thought to prevent elongation of the shorter stereocilia rows during this final phase of hair bundle maturation [44], and ELMOD1 may be involved in regulating the final length of the tallest row of stereocilia. An uncoupling of actin polymerization and plasma membrane turnover rates may underlie the stereocilia fusions observed in inner hair cell bundles of *Elmod1* mutant mice, whereby the plasma membrane is unable to fully form along the entire lengths of individual, rapidly expanding stereocilia.

Outer hair cells mature earlier and do not undergo the final rapid burst of stereocilia elongation characteristic of inner hair cells [45], and this difference in maturation pattern may relate to the different inner and outer hair cell phenotypes observed in *Elmod1* mutant mice (Figure 2). The hair bundles of outer hair cells, though not elongated, show other abnormalities in *Elmod1* mutant mice. Bundles appear normal at P0, but by P15 stereocilia in the region adjacent to the site of the kinocilium have degenerated in all outer hair cells of mutant mice. Stereocilia near the kinocilium are the first to develop, and they grow into the longest stereocilia [45]; however, it is not clear how these characteristics relate to the localized loss of stereocilia at the bundle vertex in outer but not inner hair cells.

Kinocilia of both outer and inner cochlear hair cells in *Elmod1* mutant mice appear normal and disappear as expected by P15, and hair bundle polarity is maintained throughout development. ELMOD1, therefore, is not involved in mechanisms regulating ciliogenesis or planar cell polarity.

### ELMOD1, small GTPases, and actin dynamics of stereocilia

In mammalian cells, ELMO1 forms a tertiary complex with RhoG and Dock180 to induce activation of Rac1 in a signaling cascade that mediates cell surface morphology [23]. Indirect evidence suggests that small GTPases like RhoG and Rac1 also may regulate actin cytoskeleton dynamics in hair cell stereocilia [11], [12], but which GTPases are active at the hair cell surface and the molecules that control their timing and localization are unknown. We hypothesize that ELMOD1 functions to mediate the activation or inactivation of particular small regulatory GTPases in hair cells and that perturbation of this function is responsible for the altered hair bundle morphology seen in *rda* mutant mice, in which inner hair cell stereocilia are greatly elongated and fused.

Dock4, a member of the Dock180 family, has been shown to form complexes with ELMO proteins to activate Rac1 and regulate such processes as cell migration [18] and neuronal dendrite development [46]. Dock4 has been localized to inner ear stereocilia [47], suggesting that a GTPase-DOCK4-ELMO signaling pathway may be involved in regulating the cytoskeletal dynamics of hair cells. ELMOD1, however, does not have the 200 amino acid C-terminus (plekstrin homology) characteristic of ELMO proteins 1–3 [22], and this C-terminus is the region shown to form a complex with DOCK180 [48]. ELMOD1, therefore, is not likely to interact with Dock4 and act as a GEF for Rac-mediated actin regulation in hair cell stereocilia.

ERM proteins (ezrin, radixin, moesin) have been shown to interact with ELMO1 to promote RhoG-dependent apical cytoskeleton rearrangements in epithelial cells [49], [50]. Radixin [51], [52] and the ERM-interacting protein CLIC5 [53] are expressed in hair cell stereocilia and loss of these proteins causes deafness, suggesting that an ELMO-ERM interaction may mediate actin cytoskeleton organization in hair cell stereocilia. The ERM binding domain on ELMO1 was mapped to the N-terminal 280 amino acids [49]. The N-terminal region of ELMOD proteins, however, does not have this N-terminal armadillo-like domain; therefore, ELMOD1 is not expected to bind with ERM proteins.

Recent evidence indicates that ELMO-family proteins may perform functions other than acting as GEFs to promote RAC-activation via their interaction with DOCK proteins. ELMOD1 and ELMOD2 have been shown to function as GAPs to inactivate members of the ADP-ribosylation factor (Arf) and ADP-ribosylation factor-like (Arl) families of small regulatory GTPases [22]. Arf and Arl GTPases can regulate downstream activation of Rac1 and have been implicated in the regulation of membrane trafficking and the actin cytoskeleton [10], [54], [55]. Phosphoinositides are important regulators of actin cytoskeletal organization [56], [57], [58], [59], and Arf-family GTPases and their effectors have been shown to be important mediators of this function [60], [61]. For example, Arf6 localizes to the plasma membrane and facilitates actin cytoskeletal rearrangements by activating type 1 phosphatidylinositol 4-phosphate 5-kinase (PIP-5 kinase) to generate phosphatidylinositol-4,5-biphosphate (PIP2) [62].

Protein tyrosine phosphatase receptor Q (PTPRQ) is a phosphotidylinositol phosphatase that that can regulate the phosphoinositide content of membranes [63], [64], and PTPRQ is required for normal cochlear hair bundle development [41]. Mice deficient in PTPRQ exhibit elongations of inner hair cell stereocilia [42]. The similarity of hair bundle phenotypes of *Ptprq* and *Elmod1* mutant mice suggests the possibility that ELMOD1 may be involved in PIP2 regulation of the actin cytoskeleton during hair bundle maturation, perhaps acting as a GAP for an Arf-family GTPase that mediates PIP2 production in hair cell membranes. Similar Arf-phosphoinositide interactions are known to influence cytoskeleton organization in other cell types [10], [62].

Elmo-related proteins have both GEF and GAP promoting activities on a variety of small GTPases. In the amoeba *Dictyostelium*, an Elmo-related protein (ElmoA) inhibits actin polymerization either as a GAP or by interfering with localized GEF activity [21], [65]. Cells lacking ElmoA display increased pseudopod formation and excessive F-actin polymerization within pseudopods. ElmoA is most closely related to the ELMOD1 and ELMOD2 members of the mammalian ELMO family [21], suggesting that ELMOD1 may also function as a negative regulator of actin polymerization in mammalian hair cell stereocilia. ELMOD1 is known to have GAP activity on Arf family GTPases [22] and may function as a GAP to deactivate an inner ear GTPase that specifically promotes the P7 to P13 rapid elongation of the tall stereocilia row of cochlear inner hair cells [44]. Loss of ELMOD1 thus would result in continued, excessive elongation, consistent with the striking increase in stereocilia length observed between P10 and P15 in inner hair cells of ELMOD1 deficient mice. The *Elmod1* mutant mouse model presented here will provide a means to test these proposed molecular pathways and functions.

## Materials and Methods

### Mouse strains and husbandry

Two independently arising recessive mutations named roundabout (*rda*) and roundabout-2 Jackson (*rda^2J^*) were discovered and are being maintained at The Jackson Laboratory (Bar Harbor, Maine). The *rda* mutation occurred in a C57BL/6J (B6) colony of mice, and the *rda^2J^* mutation occurred in a B10.BR-*H2^k^H2-T18^a^*/SgSnJ colony. The *rda^2J^* mutation was subsequently made congenic on a B6 strain. Mice with these two *Elmod1* mutations are available from The Jackson Laboratory (http://jaxmice.jax.org/index.html); the strain carrying the *rda* mutation is designated C57BL/6J-*Elmod1^rda^*/JKjn (Stock #2745), and the strain carrying the *rda^2J^* mutation is designated B6.Cg-*Elmod1^rda-2J^*/JKjn (Stock #6018). Experimental mice were housed in the Research Animal Facility of The Jackson Laboratory, and all procedures involving their use were approved by the Institutional Animal Care and Use Committee. The Jackson Laboratory is accredited by the American Association for the Accreditation of Laboratory Animal Care.

### Auditory-evoked brainstem response (ABR)

Hearing in mice was assessed by ABR threshold analysis, as previously described [66]. Mice were anesthetized with an intraperitoneal injection of tribromoethanol (0.2 ml of 12.5 mg/ml stock per 10 g of body weight), and then placed on a heating pad in a sound-attenuating chamber. Needle electrodes were placed just under the skin, with the active electrode placed between the ears just above the vertex of the skull, the ground electrode between the eyes, and the reference electrode underneath the left ear. High-frequency transducers were placed just inside the ear canal and computer-generated sound stimuli were presented at defined intervals. Thresholds were determined 8-, 16-, and 32-kHz pure-tone stimuli by increasing the sound pressure level (SPL) in 10-dB increments followed by 5-dB increases and decreases to determine the lowest level at which a distinct ABR wave pattern could be recognized. Stimulus presentation and data acquisition were performed using the Smart EP evoked potential system (Intelligent Hearing Systems, Miami, FL).

### Histopathology and scanning electron microscopy

Anesthetized mice were perfused through the left ventricle of the heart with phosphate-buffered saline (PBS) followed by Bouin's fixative. For microscopic analysis of cross-sections, inner ears from mutant and control mice were dissected, perfused with Bouin's fixative, immersed in the same solution for 24 to 48 hours, decalcified with Cal-EX solution for 6 hours, and embedded in paraffin. Sections (4 µm) were cut, mounted on glass slides, and counterstained in hematoxylin/eosin (H&E).

Scanning electron microscopy of inner ear organs was performed essentially as described (Furness and Hackney, 1986). Inner ears were dissected out of the skull, fixed in 2.5% glutaraldehyde in 0.1 M cacodylate buffer for 3–4 h at 4°C, and then washed three to four times in 0.1 M phosphate buffer. Hair cells of the organ of Corti were exposed by carefully dissecting away the overlying bone and membrane. Mouse cochleas were processed in osmium tetroxide-thiocarbohydrazide (OTOTO), dehydrated with ethanol, and critical-point dried with hexamethyldisilazane (Electron Microscopy Sciences, Hatfield, PA). Samples were mounted onto aluminum stubs and sputter-coated to produce a 10–15-nm gold-palladium coat. Samples were examined at 20 kV with a Hitachi 3000N VP scanning electron microscope.

### Genetic mapping and progeny testing

Genomic DNA for genotyping mice was rapidly prepared from tail tips by the hot sodium hydroxide and Tris (Hot SHOT) procedure [67]. A pooled DNA strategy using microsatellite markers [68] was used to initially localize the *rda* and *rda^2J^* mutations to Chr 9. Individual DNA samples from linkage mice were subsequently genotyped for multiple microsatellite markers and SNPs located on Chr 9. PCR was performed in a Bio-Rad PTC-200 Peltier Thermal Cycler. Amplification consisted of an initial denaturation at 97°C for 30 s followed by 40 cycles, each consisting of 94°C for 30 s (denaturation), 60°C for 30 s (annealing), and 72°C for 30 s for the first cycle and then increasing by 1 s for each succeeding cycle (extension). After amplification, the product was incubated for an additional 10 min at 72°C (final extension). PCR products were separated on 2.5% SeaKem gels (Lonza, Rockland, ME) and visualized with ethidium bromide.

For progeny testing, each recombinant mouse was mated with an *rda/rda* mutant mouse, and progeny were tested for hearing by ABR. If any progeny exhibited elevated ABR thresholds, the genotype of the recombinant parent was classified as +/*rda*; if at least 10 progeny (from two or more litters) were examined but none showed elevated thresholds, the recombinant parent was classified as +/+.

### Candidate gene sequence analysis

High quality genomic DNA for candidate gene sequencing and Southern blot analysis was prepared from mouse spleens by standard phenol/chloroform extraction and ethanol precipitation methods. To sequence cDNA and assess gene expression, total RNA from individual mouse tissues was isolated with Trizol reagent following the protocol of the manufacturer (Life Technologies Corporation, Grand Island, NY). Mouse cDNA samples were synthesized with the SuperScript First-Strand Synthesis System (Life Technologies).

PCR for comparative DNA sequence analysis was performed according to the conditions described above for linkage mapping. Primers for genotyping and amplifying portions of the mouse *Elmod1* gene for sequence comparisons were designed using Primer3 (http://primer3.sourceforge.net/) and synthesized by Integrated DNA Technologies (Coralville, IA, USA). Primer sequences for cDNA amplification are given in Table S2, and those for genomic DNA amplification are given in Table S3. PCR products were purified with the QIAquick PCR Purification Kit (Qiagen Inc., Valencia, CA). Primers used for DNA amplification were also used for DNA sequencing on an Applied Biosystems 3700 DNA Sequencer with an optimized Big Dye Terminator Cycle Sequencing method.

### Southern and northern blot analyses

For Southern and northern blot analysis, DNA probes for specific regions of the *Elmod1* gene were generated by PCR amplification of genomic DNA or brain-derived cDNA from C57BL/6J mice with the primers listed in Tables S2 and S3. The PCR fragments were separated on agarose gels, extracted and purified with the QIAquick Gel Extraction Kit (Qiagen, Inc., Valencia, CA, USA). Preparation of Southern and northern blots, probe labeling, and hybridization methods followed previously described standard procedures [69]. Commercially prepared northern blots of mouse embryos and multiple adult tissues (Clontech Laboratories, Inc., Mountain View, CA) were also analyzed. For Southern blots, 20 mg of genomic DNA, predigested with a restriction endonuclease, was loaded per lane on an 0.8% agarose gel and electrophoresed in TAE buffer at 2 V/cm for 12 hours. For northern blots, 15 mg of total RNA was loaded per lane on a 0.8% agarose gel with 2.2 M formaldehyde, electrophoresed in 1× MOPS buffer at 3 V/cm for 4 hr. DNA from Southern blots and RNA from northern blots were transferred to positvely charged nylon membranes by vacuum in 10× SSC and UV cross-linked to the membranes. Probes used for blot hybridizations were labeled with ^32^P by the random primer method.

### Gene expression analysis by RT-PCR and 5′RACE

Multiple tissues from adult wildtype mice were screened for the presence of *Elmod1* transcripts by RT-PCR using the wex3F and wex7R primers listed in Table S2. *Elmod1* transcripts from brain-derived cDNAs of wildtype mice and *rda^2J^* mutant mice were further analyzed with the multiple primer pairs listed in Table S2. Total RNA and cDNAs were prepared as described above for candidate gene sequence analysis.

For the Rapid Amplification of cDNA Ends (RACE), we used a commercial kit (FirstChoice RACE-Ready cDNA, Life Technologies, Grand Island, NY) and total RNA from mouse brain tissue. This procedure uses RNA ligase mediated RACE to amplify cDNA only from full-length capped mRNA. Reactions were performed according to manufacturer's specifications with *Elmod1* gene-specific primers given in Table S2 (wex1F, Wex2R1, wex2R2).

### Antibody preparations and western blot analysis

The complete protein coding sequence of *Elmod1* was cloned into the 2.1 TOPO vector using the TOPO TA Cloning Kit (Life Technologies, Grand Island, NY). The resulting plasmid was sent to Proteintech Group, Inc (Chicago, IL) where it was cloned into the expression vector pGEX4T-1 for protein production. Purified ELMOD1 protein was then injected into rabbits for polyclonal antibody production. High titer antiserum was collected and affinity purified.

Western blots were performed as described previously [70]. Whole brains were homogenized in lysis buffer as described. Blots were probed with affinity-purified rabbit anti-ELMOD1 antisera (1∶500) followed by anti-rabbit IgG, horseradish peroxidase (HRP)-linked F(ab′)2 fragments from donkey (1∶50,000; GE Lifesciences, Piscataway, NJ). ECL Plus western blotting detection reagents (GE Lifesciences) were used for enhanced chemiluminescent detection of specifically bound antibody by exposure to audioradiograph film (Eastman Kodak Co., Rochester, NY).

### DIG-labeled riboprobes and mRNA *in situ* hybridization

DNA sequences of primers for amplifying a 768 bp fragment of *Elmod1* cDNA from brain tissue were obtained from the Eurexpress Transcriptome Atlas Database for Mouse Embryo (www.eurexpress.org, Template ID T39634) [71]. The sequence for the SP6 promoter was added as a 5′overhang to the reverse primer so that the antisense probe could be generated directly from the PCR product. A digoxigenin-11-UTP (DIG) labeled antisense riboprobe was produced from the purified PCR product by in vitro transcription according to the manufacturer's instructions (Roche Applied Science, Indianapolis, IN).

*In situ* hybridization was performed as described [72] with a the following modifications. Tissue was fixed overnight at 4°C in 4% paraformaldehyde. Sections were mounted on Superfrost plus slides (Fisher Scientific, Suwanee, GA). Slides were air dried at room temperature for 1–2 hours, followed by fixation in 4% parafomaldehyde for 10 minutes. Digestion was carried out for 10 min at room temperature in buffer consisting of 50 mM Tris, pH 7.5, 5 mM EDTA, and 1 µg/ml proteinase K followed by fixation in 4% paraformaldehyde for 5 min. Hybridization was performed at 65° C overnight in buffer consisting of 50% deionized formamide, 0.3 M NaCl, 20 mM Tris, pH 8.0, 5 mM EDTA, pH 8.0, 10% dextran sulfate, 1× Denhardt's solution, 0.5 mg/ml tRNA from baker's yeast (Sigma, St. Louis, MO), and 500 ng/ml of the denatured DIG labeled probe. Washes were also carried out at 65° C. Following immunological detection [72], slides were mounted with VECTASHIELD mounting medium (Vector Laboratories, Burlingame, CA).

## Supporting Information

Figure S1 **Analyses of** ***Elmod1*** **alternative transcripts and promoter site.** **A. Exon structure of the** ***Elmod1*** **gene and alternative splicing.** White rectangles (proportionately sized) represent individual exons of the reference sequence NM-177769 and are numbered according to their linear sequence on Chr 9. Intronic regions are not shown, but splicing events are represented as lines connecting the ends of exons. Exon regions from alternatively spliced transcripts are indicated by black rectangles. The mouse cDNA sequences AK029207, BC120566, BC144961, and BC049160 include an additional 38 bp at the 5′ end of exon 3 (indicated by black rectangle labeled “a”), presumably the result of an alternative splice acceptor site. Our analysis of PCR products from cDNA templates using exon 1 and exon 3 specific primers indicates that the shorter 146-bp exon 3 is the most predominant isoform in adult mouse brain tissue, although transcripts with the extended 184-bp exon 3 are detected in low abundance (lane 1 in panel B). Reference sequences for the human *ELMOD1* gene are derived from two transcripts: a 2990 bp isoform 1 (NM_018712), which is transcribed from 12 exons and encodes a 334 aa protein, and a 2967 bp isoform 2 (NM_001130037), which is transcribed from 11 exons and encodes a 326 aa protein. The NM_018712 isoform includes an additional, in-frame 24-bp exon that lies between exons 8 and 9 of NM_001130037 (indicated by black rectangle labeled “b”). None of the reported mouse cDNAs or ESTs includes this extra exon; however, we found evidence for it by exon-specific PCR analysis of cDNAs from mouse brain tissue, although it is much less abundant than transcripts lacking this alternative, additional exon (lane 2 in panel B). The mouse AK029207 cDNA sequence for *Elmod1* includes an alternative last exon (11A, indicated by black rectangle labeled “c” in **A**), which is different from the standard last exon (11S) of NM_177769 and most other cDNAs and spliced ESTs. We did not detect this alternative exon 11 in our PCR analyses of cDNA from mouse brain tissue using exon 10 primers and primers specific for the alternative exon 11 of AK029207 (Table S2). **B. Alternative, low abundance** ***Elmod1*** **transcripts detected in mouse brain tissue.** Lane 1 shows PCR products from adult mouse brain cDNA obtained with primers wex1F and wex3R2. These primers are specific to exons 1 and 3. The PCR product size for the predominant transcript, which contains the smaller exon 3 (146 bp), is expected to be 325 bp, whereas the product size for the alternative transcript (marked by asterisk), which contains the additional 38 bp (designated 3A in panel B) of the extended exon 3, is expected to be 363 bp. Lane 2 shows PCR products from the same cDNA obtained with primers wex7F and wex10R. These primers are specific to exons 7 and 10. The PCR product for the predominant transcript is expected to be 236 bp; whereas the less abundant PCR product (marked by asterisk) is expected to be 260 bp because the alternative transcript includes an additional 24 bp exon between the standard exons 8 and 9. The lane marked “bp” is a 100 bp size ladder with 100 bp and 500 bp positions indicated by arrows. The DNA sequences of PCR primers are given in Table S2B. **C. Transcription start site detected in mouse brain tissue.** A 5′ RACE assay with brain-derived cDNA and nested primers specific to exon 2 was used to determine the transcription start site of *Elmod1*. Lane 1 shows the 250 bp PCR product that was produced with the 5′ RACE outer primer and a gene-specific outer primer (wex2R1), and Lane 2 shows the 220 bp product that was produced with the 5′ RACE inner primer and a gene-specific inner primer (wex2R2). Lane 3 is a negative control with no cDNA, and Lane 4 is a positive control showing the 195 bp product expected with two gene-specific internal primers (wex1F and wex2R2). The DNA sequences of PCR primers are given in Table S2B. The lane marked “bp” is a 100 bp size ladder with 100 bp and 500 bp positions indicated by arrows. We identified a single transcription start site corresponding approximately to nucleotides 120–130 of NM_177769. Consistent with this result, we were unable to produce RT-PCR products with primers corresponding to cDNA sequences that are 5′ of this region.
(TIF)

Figure S2 ***In situ*** **expression of** ***Elmod1*** **mRNA in brain and eye.** Expression of *Elmod1* was detected in specific regions of the brain in wildtype (+/+) mice, including Purkinje cells (PCs) of the cerebellum (**A**). *Elmod1* expression also was detected in the hippocampus, where it was localized to the granule cell layer (Gcl) of the dentate gyrus and pyramidal neuron cell bodies (Pyr) of the cornu ammonis (**B**). Shown are brain sections of postnatal day 45 (P45) mice, but similar expression patterns were seen at P7 and P15. *Elmod1* expression was also detected in retinal ganglion cells (RGCs) in the eyes of +/+ mice at P2 (**C**) and at P15 (not shown). Mutant *rda/rda* mice served as negative controls for probe specificity, as seen by the lack of detectable *Elmod1* expression in the cerebellum of *rda/rda* mice (**D**). All panels are at the same magnification; the scale bar represents 250 micrometers.
(TIF)

Table S1 **Genetic map position of the** ***rda*** **mutation on mouse Chr 9.**
(PDF)

Table S2 **PCR primers for cDNA amplification.**
(PDF)

Table S3 **PCR primers for genomic DNA amplification.**
(PDF)
