# Supplementary figures and images for: Mutations of the Mouse ELMO Domain Containing 1 Gene (Elmod1) Link Small GTPase Signaling to Actin Cytoskeleton Dynamics in Hair Cell Stereocilia
Source: PLoS One. 2012 Apr 27;7(4):e36074. doi: 10.1371/journal.pone.0036074 (PMC3338648; doi:10.1371/journal.pone.0036074)

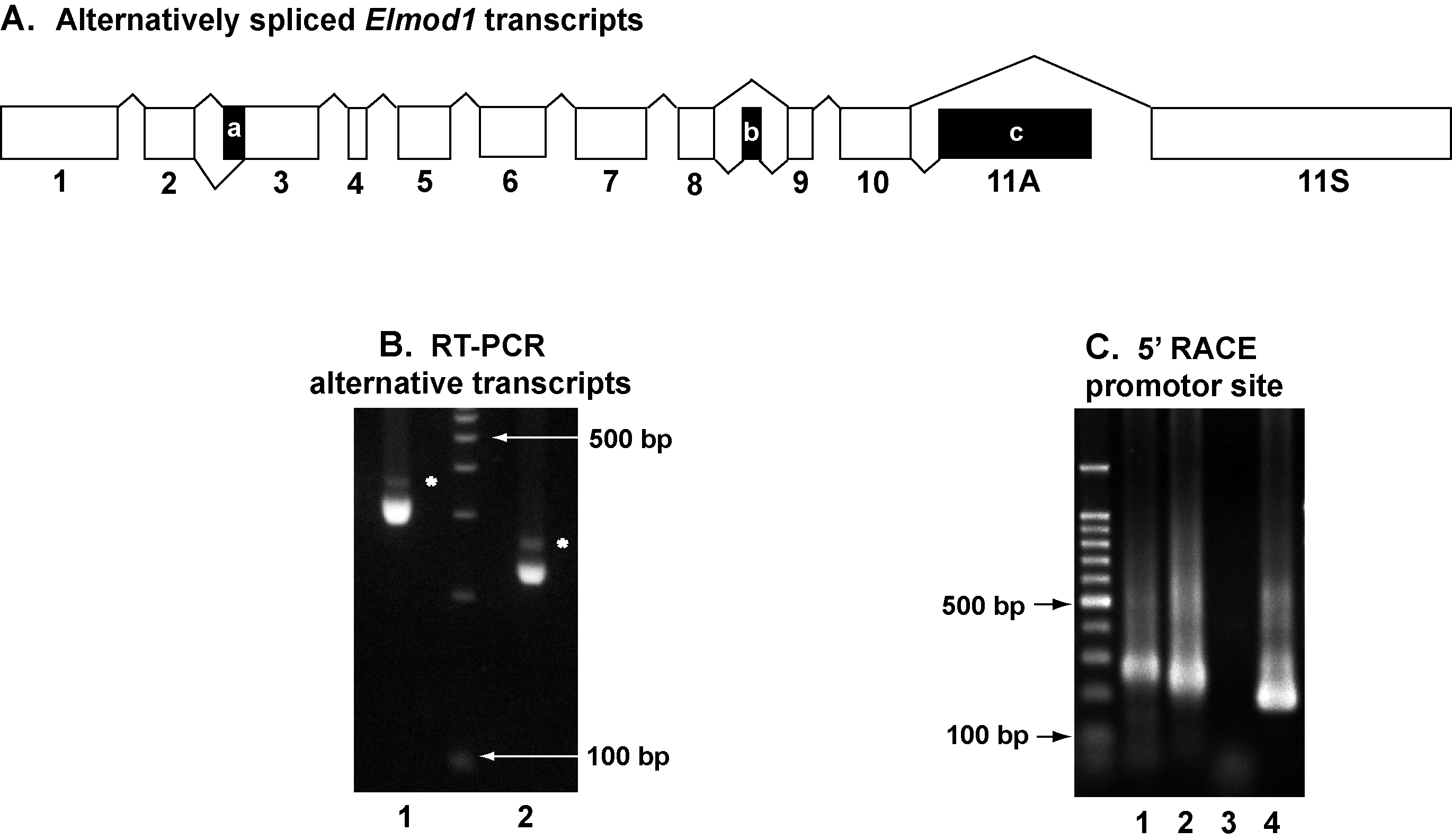

Supplement: Figure S1 — Analyses of Elmod1 alternative transcripts and promoter site. A. Exon structure of the Elmod1 gene and alternative splicing. White rectangles (proportionately sized) represent individual exons of the reference sequence NM-177769 and are numbered according to their linear sequence on Chr 9. Intronic regions are not shown, but splicing events are represented as lines connecting the ends of exons. Exon regions from alternatively spliced transcripts are indicated by black rectangles. The mouse cDNA sequences AK029207, BC120566, BC144961, and BC049160 include an additional 38 bp at the 5′ end of exon 3 (indicated by black rectangle labeled “a”), presumably the result of an alternative splice acceptor site. Our analysis of PCR products from cDNA templates using exon 1 and exon 3 specific primers indicates that the shorter 146-bp exon 3 is the most predominant isoform in adult mouse brain tissue, although transcripts with the extended 184-bp exon 3 are detected in low abundance (lane 1 in panel B). Reference sequences for the human ELMOD1 gene are derived from two transcripts: a 2990 bp isoform 1 (NM_018712), which is transcribed from 12 exons and encodes a 334 aa protein, and a 2967 bp isoform 2 (NM_001130037), which is transcribed from 11 exons and encodes a 326 aa protein. The NM_018712 isoform includes an additional, in-frame 24-bp exon that lies between exons 8 and 9 of NM_001130037 (indicated by black rectangle labeled “b”). None of the reported mouse cDNAs or ESTs includes this extra exon; however, we found evidence for it by exon-specific PCR analysis of cDNAs from mouse brain tissue, although it is much less abundant than transcripts lacking this alternative, additional exon (lane 2 in panel B). The mouse AK029207 cDNA sequence for Elmod1 includes an alternative last exon (11A, indicated by black rectangle labeled “c” in A), which is different from the standard last exon (11S) of NM_177769 and most other cDNAs and spliced ESTs. We did not detect this alternative e [file pone.0036074.s001.tif]

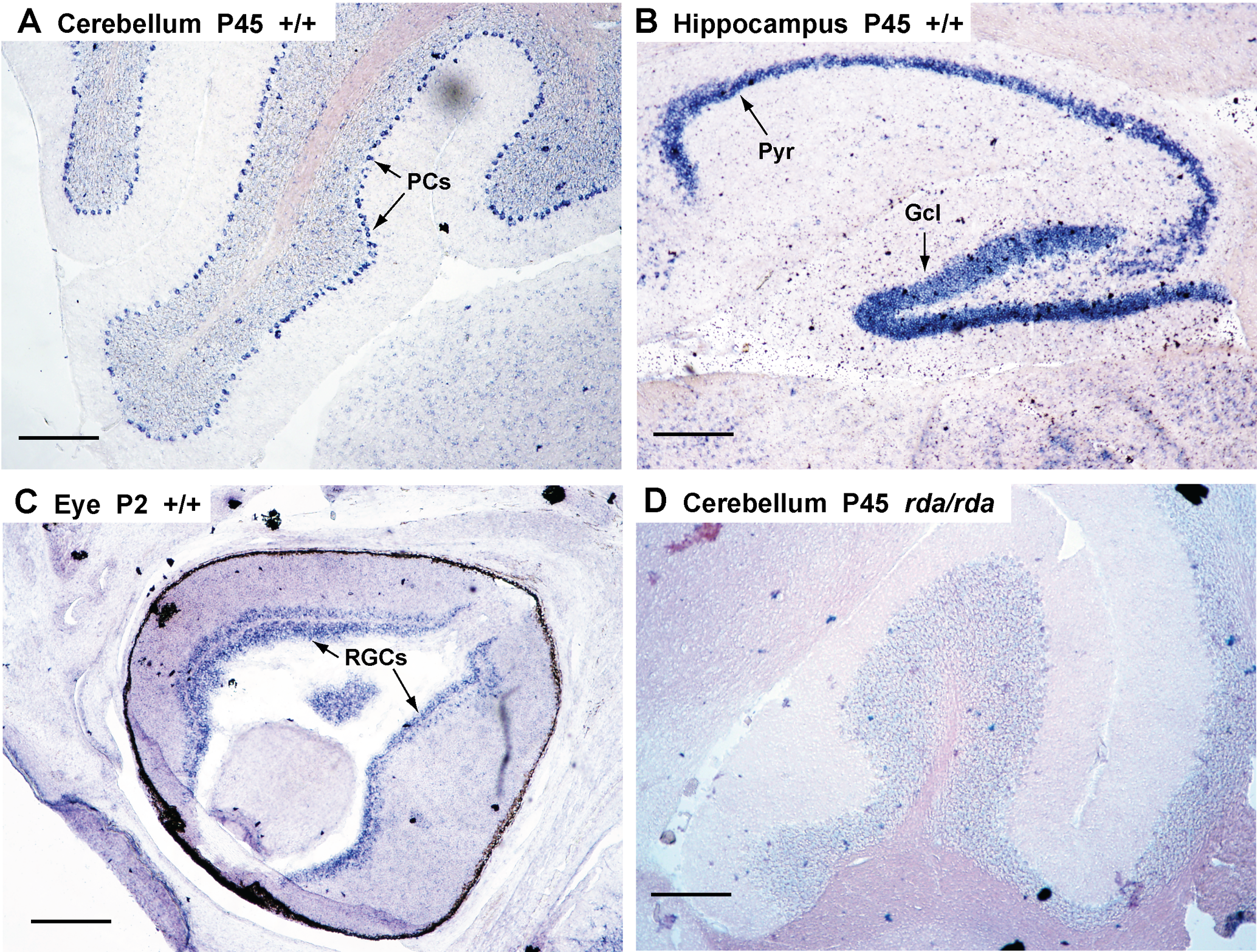

Supplement: Figure S2 — In situ expression of Elmod1 mRNA in brain and eye. Expression of Elmod1 was detected in specific regions of the brain in wildtype (+/+) mice, including Purkinje cells (PCs) of the cerebellum (A). Elmod1 expression also was detected in the hippocampus, where it was localized to the granule cell layer (Gcl) of the dentate gyrus and pyramidal neuron cell bodies (Pyr) of the cornu ammonis (B). Shown are brain sections of postnatal day 45 (P45) mice, but similar expression patterns were seen at P7 and P15. Elmod1 expression was also detected in retinal ganglion cells (RGCs) in the eyes of +/+ mice at P2 (C) and at P15 (not shown). Mutant rda/rda mice served as negative controls for probe specificity, as seen by the lack of detectable Elmod1 expression in the cerebellum of rda/rda mice (D). All panels are at the same magnification; the scale bar represents 250 micrometers. (TIF) [file pone.0036074.s002.tif]
