# Supplementary material for: Mutations of the Mouse ELMO Domain Containing 1 Gene (Elmod1) Link Small GTPase Signaling to Actin Cytoskeleton Dynamics in Hair Cell Stereocilia
Source: PLoS One. 2012 Apr 27;7(4):e36074. doi: 10.1371/journal.pone.0036074 (PMC3338648; doi:10.1371/journal.pone.0036074)
Supplement: Table S1 — Genetic map position of the rda mutation on mouse Chr 9. (PDF) [file pone.0036074.s003.pdf]

Table S1. Genetic map position of the *rda* mutation on mouse Chr 9. Shown are marker genotypes for 48 mice that were recombinant between *D9Mit231* and *D9Mit248*, out of a total of 1357 F2 mice generated from a (B6-*rda* x CAST) intercross. The closest flanking markers are highlighted in green and defined a 1.14 Mb candidate gene interval. Genotypes homozygous for CAST alleles are denoted C and highlighted in yellow. Genotypes heterozygous for B6 and CAST alelles are denoted H and highlighted in pink. Genotypes homozygous for B6 alleles are denoted B and highlighted in blue. The *rda* genotypes were determined from ABR thresholds of F2 and phenotypes of progeny test mice. The *Elmod1* gene (53.8 Mb) lies within the *rda* candidate region (53.19 – 54.33 Mb).

		Genetic markers and their Chr 9 Mb positions (NCBI Build 37)							
mouse		52.09	53.19	53.34		54.33	54.78	58.21	progeny test
ID	ABR	*D9Mit231*	*Exph5*	*Atm*	*rda*	*D9Krj4**	*D9Mit100*	*D9Mit248*	mutants/total
381	normal	H	H	H	+/*rda*	H	H	C	
402	elevated	B	B	B	*rda/rda*	B	B	H	
408	elevated	H	B	B	*rda/rda*	B	B	B	
410	elevated	H	B	B	*rda/rda*	B	B	B	
423	elevated	B	B	B	*rda/rda*	B	B	H	
442	elevated	B	B	B	*rda/rda*	B	B	H	
469	elevated	B	B	B	*rda/rda*	B	B	H	
503	elevated	B	B	B	*rda/rda*	B	H	H	
517	elevated	H	B	B	*rda/rda*	B	B	B	
558	normal	C	C	C	+/+	C	H	H	0/23
593	elevated	B	B	B	*rda/rda*	B	H	H	
613	elevated	B	B	B	*rda/rda*	B	H	H	
615	normal	H	C	C	+/+	C	C	C	0/18
633	normal	H	H	H	+/*rda*	H	H	B	
649	normal	H	H	H	+/*rda*	H	H	B	
672	normal	C	H	H	+/*rda*	H	H	H	2/7
694	normal	H	C	C	+/+	C	C	C	0/14
719	normal	C	H	H	+/*rda*	H	H	H	1/8
756	elevated	B	B	B	*rda/rda*	B	B	H	
778	elevated	H	B	B	*rda/rda*	B	B	B	
785	-	C	C	C	+/+	C	C	H	
794	elevated	H	H	B	*rda/rda*	B	B	B	
811	normal	H	H	H	+/*rda*	H	H	B	
868	-	C	C	C	+/+	C	C	H	0/15
872	-	H	H	H	+/*rda*	H	H	C	4/11
877	-	C	C	C	+/+	H	H	H	0/13
904	-	H	C	C	+/+	C	C	C	0/18
945	elevated	H	B	B	*rda/rda*	B	B	B	
999	normal	B	H	H	+/*rda*	H	H	H	
1026	-	C	H	H	+/*rda*	H	H	H	4/6
1031	normal	H	H	H	+/*rda*	H	B	B	
1034	-	H	H	H	+/*rda*	H	H	C	1/3
1053	normal	H	H	H	+/*rda*	H	H	B	
1056	-	H	H	H	+/*rda*	H	H	C	4/6
1141	-	H	C	C	+/+	C	C	C	0/16
1170	-	C	C	C	+/+	C	C	H	0/21
1191	-	C	C	C	+/+	C	C	H	
1208	normal	B	H	H	+/*rda*	H	H	H	
1211	normal	H	H	H	+/*rda*	H	H	B	
1235	-	H	C	C	+/+	C	C	C	0/21
1242	normal	H	H	H	+/*rda*	H	H	B	
1267	elevated	B	B	B	*rda/rda*	B	B	H	
1270	elevated	B	B	B	*rda/rda*	B	B	H	
1283	elevated	B	B	B	*rda/rda*	B	B	H	
1300	elevated	B	B	B	*rda/rda*	B	B	H	
1471	normal	H	H	H	+/*rda*	H	B	B	
1479	normal	H	H	H	+/*rda*	H	B	B	
1480	normal	H	H	H	+/*rda*	H	B	B	

* CA repeat (Chr 9:54325790-54325843)
