# Supplementary material for: Mutations of the Mouse ELMO Domain Containing 1 Gene (Elmod1) Link Small GTPase Signaling to Actin Cytoskeleton Dynamics in Hair Cell Stereocilia
Source: PLoS One. 2012 Apr 27;7(4):e36074. doi: 10.1371/journal.pone.0036074 (PMC3338648; doi:10.1371/journal.pone.0036074)
Supplement: Table S2 — PCR primers for cDNA amplification. (PDF) [file pone.0036074.s004.pdf]

Table S2. PCR primers for cDNA amplification.

name	forward primer sequence	name	reverse primer sequence
**A. To sequence *Elmod1* cDNA and produce Southern and northern blot probes.**			
wex1F	CTCTGTCCAGCATCCGCTC	wex3R	TTCTGGAAGCTCCAGGTTTG
primers within exons 1 and 3, 367 bp product (additional 973 bp product in *rda-2J*)			
wex1F	CTCTGTCCAGCATCCGCTC	wex11R1	ATCTGGGTTCTGCAGCTGTT
primers within exons 1 and 11, 1147 bp product (1753 bp product in *rda-2J*)			
wex3F	CGCTGCATGAAGTTTGTGAT	wex7R	AGTCCCAGAAGACCCATTCC
primers within exons 3 and 7, 479 bp product (additional 1085 bp product in *rda-2J*)			
	Southern and northern blot probe		
wex7F	TTTCGAAGCAGTGGTGTGAG	wex11R1	ATCTGGGTTCTGCAGCTGTT
primers within exons 7 and 11, 466 bp product (additional 1072 bp product in *rda-2J*)			
	Southern blot probe		
wex8F	TCCTCTCCGACTCTGTCCAT	wex11R2	CTTGTGAAACTCGTGCATCAA
primers within exons 8 and 11, 242 bp product (additional 848 bp product in *rda-2J*)			
Wex9F	TGGGAAAAGAAAAAGATGGA	wex11R3	ATTTCTCCCTCACGCGATTA
primers within exons 9 and 11, 250 bp product			
wex10F	CTTTGCAATTGTGGGCATC	wex11R1	ATCTGGGTTCTGCAGCTGTT
primers within exons 10 and 11, 249 bp product			
wex11F	GAAATTCCGCAAGAGGATCA	wex11R4	GCAGCAGTCTTCGTGAGACA
primers both within exon 11, 556 bp product			
**B. To analyze the *Elmod1* promoter site and alternative transcripts.**			
**primers for 5' RACE to analyze promotor site:**			
wex1F	CTCTGTCCAGCATCCGCTC	wex2R2	GGATGCTACGCCAACTGTCT
control forward primer in exon 1		inner reverse primer in exon 2	
		wex2R1	ATTTTGCAGGCGTTTGACTC
		outer reverse primer in exon 2	
**primers to analyze alternative 5' splicing of exon 3:**			
wex1F	CTCTGTCCAGCATCCGCTC	wex3R	TTCTGGAAGCTCCAGGTTTG
primers within exons 1 and 3, predominantly 367 bp product, small amount of alternative 405 bp product			
wex1F	CTCTGTCCAGCATCCGCTC	wex3R2	GTTCACACCTTCCGGTGAGT
primers within exons 1 and 3, predominantly 325 bp product, small amount of alternative 363 bp product			
wexS2/3F	CTTCCTGAGGATGTTGATCCAG	wex7R	AGTCCCAGAAGACCCATTCC
primers for standard exon 2/3 splice sequence and exon 7, 534 bp product			
wexA2/3F	ATGAAGCACTTCCTGAGCTTTC	wex7R	AGTCCCAGAAGACCCATTCC
primers for alternative exon 2/3 splice sequence and exon 7, 580 bp product–verified alternative splice			
**primers to analyze presence of an additional exon between standard exons 8 and 9:**			
wex7F	TTTCGAAGCAGTGGTGTGAG	wex10R	GATGCCCACAATTGCAAAG
primers within exons 7 and 10, predominantly 236 bp product, small amount of 260 bp alternative product			
wex7F	TTTCGAAGCAGTGGTGTGAG	wexA8.5R	TGATATCCCTGCATTTTGGA
primers within exon 7 and alternative exon, 168 bp product–verified presence of alternative exon			
**primers to analyze presence of an alternative last exon:**			
wex7F	TTTCGAAGCAGTGGTGTGAG	wexA11R1	GGTCTTGGGGATCAAATTCA
primers within exons 10 and alternate exon 11, expect 436 bp product if transcribed–not detected			
wex7F	TTTCGAAGCAGTGGTGTGAG	wexA11R2	CACAGACCTTGGTTTGGTAGA
primers within exons 10 and alternate exon 11, expect 580 bp product if transcribed–not detected			
