# Supplementary material for: Mutations of the Mouse ELMO Domain Containing 1 Gene (Elmod1) Link Small GTPase Signaling to Actin Cytoskeleton Dynamics in Hair Cell Stereocilia
Source: PLoS One. 2012 Apr 27;7(4):e36074. doi: 10.1371/journal.pone.0036074 (PMC3338648; doi:10.1371/journal.pone.0036074)
Supplement: Table S3 — PCR primers for genomic DNA amplification. (PDF) [file pone.0036074.s005.pdf]

Table S3. PCR primers for genomic DNA amplification.

name	forward primer sequence	name	reverse primer sequence
**A. To analyze *Elmod1* exons and splice sites.**			
ex1F	CCCTTTCTTCCCACATTCTG	ex1R	ATTCACCCTTGGCTCCTCTA
primers flanking exon 1, 435 bp product (deleted in *rda*)			
ex2F	ACATGGAGCATGCTGTCGTA	ex2R	GCTGAGGACCAGAGGAAAGA
primers flanking exon 2, 269 bp product (deleted in *rda*)			
ex3F	GGCTTCTTTTCTTGCTGGTG	ex3R	AATGGGCATTGCAACTTCA
primers flanking exon 3, 293 bp product (deleted in *rda*)			
ex3F	GGCTTCTTTTCTTGCTGGTG	ex4R	CCTGGGCATCAAACTACCTT
primers flanking exons 3 and 4, 498 bp product (deleted in *rda*)<br>Southern blot probe			
ex4F	CAATGCCCATTTCCGTAAGTA	ex4R	CCTGGGCATCAAACTACCTT
primers flanking exon 4, 216 bp product (deleted in *rda*)			
ex5F	CAGAAGTGTGGCACTTGACG	ex5R	CCCATGTCCTGTATTTCTCACA
primers flanking exon 5, 230 bp product (deleted in *rda*)			
ex6F	ACAAGCATCCAGTGAGCAGA	ex6R	GAGTTGCTTGGCAGCAGTTA
primers flanking exon 6, 250 bp product			
ex7F	AGACAGGGTGCTTGTGTGTG	ex7R	GGTTCAGGGCCAACAGACTA
primers flanking exon 7, 252 bp product			
ex8F	CTACCTCTGAGTGCCAAGTGC	ex8R	TCCAAAAAGTTTAAATCCAACCTC
primers flanking exon 8, 200 bp product			
ex9F	CATTCAAGGGGAGCAAGAAC	ex9R	GGAGTTACCATGTGGGTGCT
primers flanking exon 9, 208 bp product			
ex10F	TGGAGTGCTTCTATCCCATGT	ex10R	TCAGCTGTGACCCTTGTCTG
primers flanking exon 10, 259 bp product			
ex11F	CTCATCCACTGAGGATTGGAA	ex11R	GGATGACAATGAGGCAGTGTT
forward primer flanking exon 11 and reverse primer within exon 11, 297 bp product			
**B. To characterize the *rda* deletion.**			
fl-DelF	TCTTGGCACAAGAAGGTTCC	fl-DelR	AACATCTGCGTGAGGGAAAG
primers flanking the *rda* deletion, 963 bp product in *rda* DNA (includes 757 bp insertion)<br>no product in wild-type DNA (region too large to amplify)			
***rda* mutation genotyping - three primer assay: DelF + InsF + fl-DelR:**			
DelF	TGTTTGCATGAGGACTTCAGA	fl-DelR	AACATCTGCGTGAGGGAAAG
primers within deletion and flanking 3' end of deletion, 218 bp in wild-type DNA, no product in *rda* DNA			
InsF	ATTCAGGATGCCTTTGCTGT	fl-DelR	AACATCTGCGTGAGGGAAAG
primers within insertion and flanking 3' end of deletion, 190 bp in *rda* DNA, no product in wild-type DNA			
